# Airway recanalization of bronchial metastasis and obstruction by smooth muscle tumour of uncertain malignant potential by a silicone OKI‐stent **™**

**DOI:** 10.1002/rcr2.798

**Published:** 2021-06-03

**Authors:** Atsushi Torii, Hideo Saka, Rieko Nishimura, Yushi Saito, Masahide Oki

**Affiliations:** ^1^ Department of Respiratory Medicine National Hospital Organization Nagoya Medical Center Nagoya Japan; ^2^ Department of Respiratory Medicine Matsunami General Hospital Gifu Japan; ^3^ Department of Pathology National Hospital Organization Nagoya Medical Center Nagoya Japan; ^4^ Department of Respiratory Surgery Toyota Memorial Hospital Toyota Japan

**Keywords:** Airway recanalization, airway stent, bronchial metastasis, dedicated bifurcated silicone stent, STUMP

## Abstract

There is no standard treatment for smooth muscle tumour of uncertain malignant potential (STUMP) but it usually has a good prognosis. Airway stenting is performed to manage central airway patency. In the present case, it was no treatment for STUMP, but performance status was good, so airway stenting was performed.

## Graphical abstract

Smooth muscle tumours of uncertain malignant potential (STUMP) are rare and there is no standard treatment. We present a patient diagnosed with pulmonary metastasis of STUMP who did not respond to treatment. Airway stenting was performed to manage airway patency and improve respiratory symptoms.

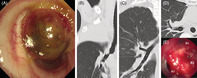

## Clinical Image

**Figure 1:**
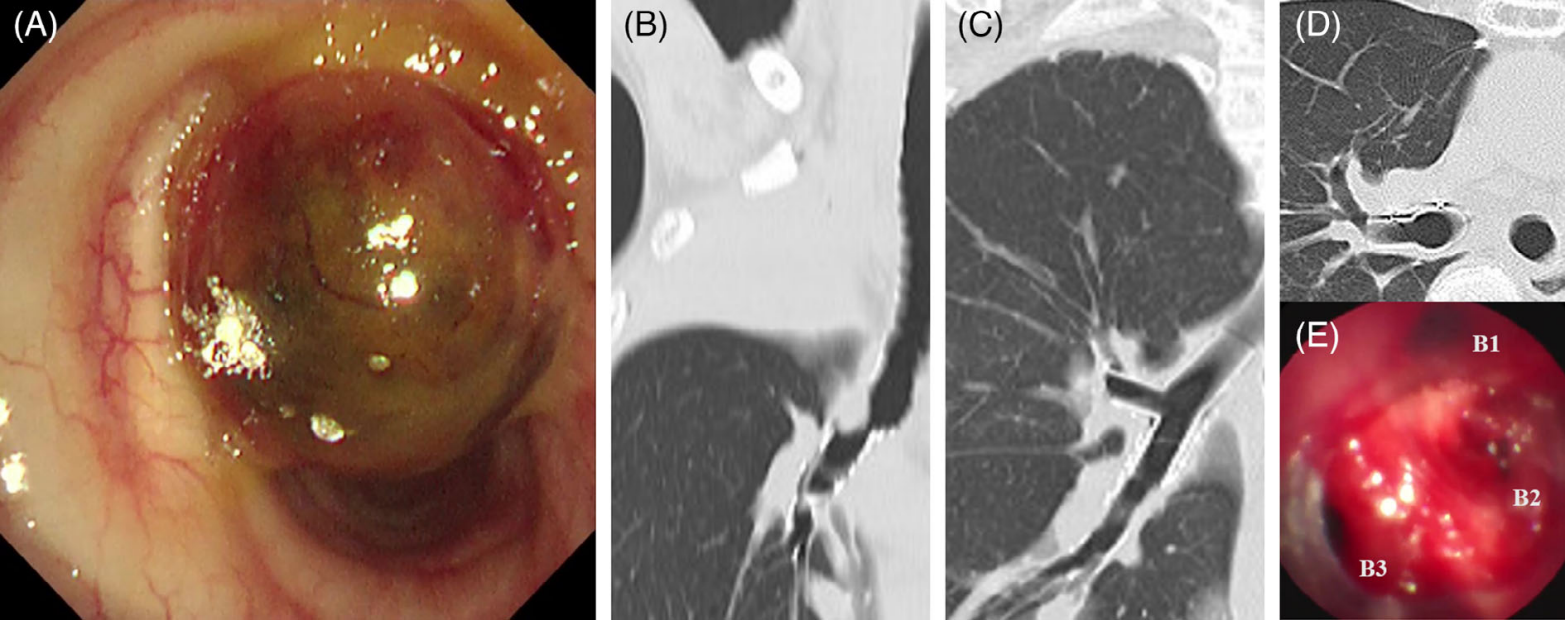
(A) Bronchoscopy revealed a large, red, smooth surface lesion with irregular vascularization and necrosis. (B) Chest computed tomography showed a lesion in the right main bronchus and right upper lobe atelectasis. (C, D) Chest computed tomography indicated that the OKI‐stent™ (the outer diameter and length of limbs for the right main stem bronchus, bronchus intermedius, and right upper lobe bronchus were 13–10–9 and 30–20–10 mm) was placed successfully in the right main bronchus and the upper lobe bronchus. The atelectasis was resolved. (E) The stent was extended near B2, where the tumour had originated, to avoid reinvasion.

**Figure 2:**
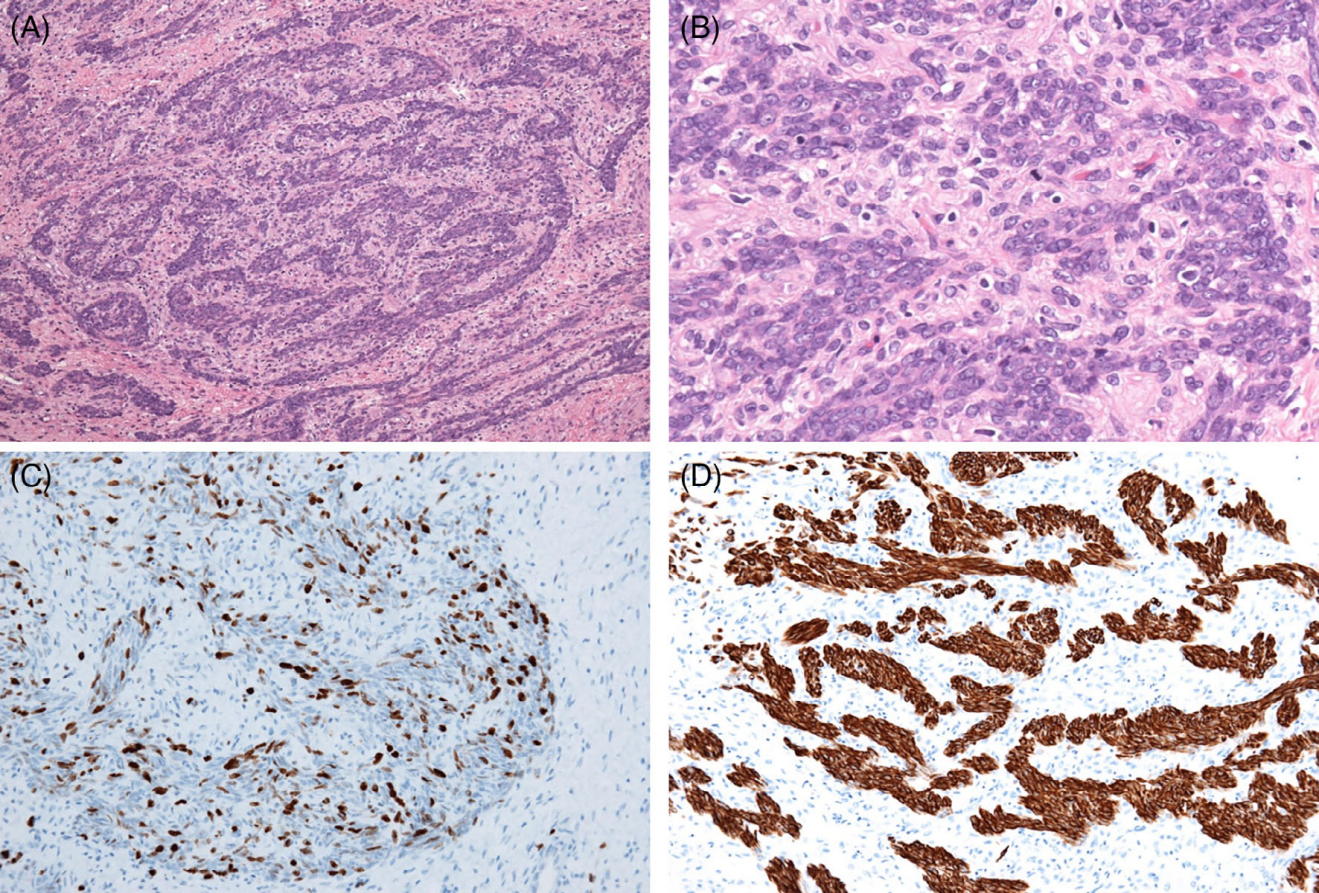
(A) Microscopy depicted proliferating monoclonal smooth muscle tumour cells with oval nuclei associated with necrosis and bleeding (haematoxylin and eosin staining, 100× magnification). (B) The mitotic index was 4/10 HP (haematoxylin and eosin staining, 400× magnification). (C) MIB‐1 index was less than 20%. (D) Immunohistochemical staining for desmin was positive.

Smooth muscle tumours of uncertain malignant potential (STUMP) are rare. The World Health Organization classification indicates as a uterine smooth muscle tumour diagnosed between benign and malignant criteria. There is no standard treatment, it has a high recurrence rate, and the median survival time is reportedly 61.5 months [1]. We present the case of a 68‐year‐old women who underwent surgery for a uterine tumour in January 2010, with lesions in right lung S3 and S4 that were detected in March 2013. The diagnosis was metastasis of STUMP. Hormone, chemotherapy, and radiation therapies were administered, but they were ineffective. In June 2018, she complained of breathing difficulty and chest computed tomography scan depicted right main bronchus constriction and right upper lobe atelectasis (Fig. 1A). She was referred to our institution and rigid bronchoscopic intervention was performed. A smooth round surface lesion with some vessels and necrosis was detected (Fig. 1A) and resected via cryorecanalization with minimal bleeding. A dedicated bifurcated silicone stent was placed in the right main bronchus to prevent reinvasion because only the lesion had been growing rapidly. Atelectasis and respiratory symptoms improved (Fig. 1C–E) and the pathological diagnosis was metastasis of STUMP (Fig. 2). After 10 months, she died in relation to STUMP.

### Disclosure Statement

Appropriate written informed consent was obtained for publication of this case report and accompanying images.

### Author Contribution Statement

Atsushi Torii contributed substantially to the writing of the manuscript. Hideo Saka, Yushi Saito, and Masahide Oki contributed substantially to critical review, and Rieko Nishimura contributed to the pathological diagnosis. All authors reviewed and approved the final version of the manuscript.

## References

[1] Bacanakgil BH , Deveci M , Karabuk F , et al. 2017. Uterine smooth muscle tumor of uncertain malignant potential: clinicopathologic‐sonographic characteristics, follow‐up and recurrence. World J. Oncol. 8:76–80. 29147439 10.14740/wjon1031w PMC5650001

